# Case of Masked Double Tertian

**Published:** 1829-07-01

**Authors:** 


### XXXII.

### CASE OF MASKED DOUBLE TERTIAN FEVER. By M. GUIBERT.

Mad. G——, aged 20 years, and of a nervous temperament, had been subject, for five weeks, to copious perspirations, which came on about three o'clock each morning, lasting till seven or eight o'clock. The urine, all this time, was high-coloured, and deposited a sediment. In all other respects the lady appeared in good health. The night of the 2d December passed without the usual perspiration, and Mad. G. rose in the morning, complaining of great weakness and tendency to faint. She was very pallid, her voice extremely feeble—in short, she appeared in a dying state, both to herself and those around her. Nevertheless the respiration and pulse were unaffected. She took some nourishment and a glass of wine, by which she was somewhat recruited—and in the evening she was almost well. She passed a good night, and the perspiration returned at the usual hour next morning, after which, she got up and breakfasted in her usual health. At 11 o'clock on that day (3d of December) she experienced a slight malaise, which rapidly increased, and in a short time, was insupportable. The most violent pains were felt in all the limbs and in the abdomen. This latter appeared to the patient to be suddenly and greatly inflated—an icy coldness seized the body, and she was soon delirious. There was next a most violent pain over the whole surface of the head, with great heat and flushing of the face. The pulse was imperceptible at the wrist, but the carotids were felt beating. Having recovered from her delirium, the former state of despondency returned, and she declared that she was dying. She therefore assembled her parents and relations around her bed, in order to take leave of them. The pulse continued imperceptible at the wrist—the extremities were of icy coldness—there were cramps and convulsive twitchings in the legs and thighs—the respiration was oppressed—the eyes rolled in their sockets—but the head continued remarkably hot, notwithstanding the application of ices. In this state, Dr. Ratheau was called into consultation, and conceived that the disease was phrenitis. Leeches were therefore applied to the head, and sinapisms to the legs. The cephalalgia diminished—the extremities became warm—sweat broke out—and, in the course of the next morning (4th December) there was a great remission, almost amounting to an intermission. At three o'clock, p. m. there was a return of cold in the extremities, and pain and heat in the head, &c. The patient demanded leeches to the *temples* and sinapisms to the extremities. But the symptoms continued till nine o'clock in the evening, when a remission again took place, preceded by a copious sweat, and deposition in the urine. The physicians now began to suspect that they had to deal with an intermittent fever under a somewhat masked form. They therefore administered the cinchona in injection. Next day, (6th December) there was another accession, after which, the sulphate of quinine was exhibited, and the disease was stopped.—*Journal Hebdomadaire.*

When such cases as the above are narrated in print, it appears astonishing to the reader that medical men should be so blind as to overlook the intermittent character of these complaints. But daily observation presents us with examples where these characters are far less masked than in the above case, and yet where the practitioner goes on leeching, bleeding, and purging, whenever symptoms of excitement appear. The misery occasioned by such oversights is much greater than people imagine. None but those who have suffered an attack of the "FOUL FIEND," can appreciate the wretched sensations by which it is accompanied—especially when aggravated by unnecessary depletion, employed under the mistaken idea that the disease is of an inflammatory nature. We would therefore caution practitioners against blind routinism, and beg them to remember that now, as in the days of Sydenham, diseases differ in different seasons, and consequently require a modification of treatment according to the nature of each "CONSTITUTION OF THE ATMOSPHERE."

